# Why do doctors emigrate from Sri Lanka? A survey of medical undergraduates and new graduates

**DOI:** 10.1186/1756-0500-7-918

**Published:** 2014-12-16

**Authors:** Nipun Lakshitha de Silva, Keshinie Samarasekara, Chaturaka Rodrigo, Lasitha Samarakoon, Sumadhya Deepika Fernando, Senaka Rajapakse

**Affiliations:** Department of Clinical Medicine, Faculty of Medicine, University of Colombo, 25 Kynsey Road, Colombo 8, Sri Lanka; National Hospital, Colombo, Sri Lanka; Department of Parasitology, Faculty of Medicine, University of Colombo, Colombo, Sri Lanka

## Abstract

**Background:**

Migration of medical professionals is a long recognized problem in Sri Lanka, but it has not been studied in depth. Undergraduate and postgraduate medical education in Sri Lanka is state sponsored, and loss of trained personnel is a loss of investment. This study assessed the intention to migrate among medical students and newly passed out graduates from the largest medical school in Sri Lanka.

**Methods:**

A cross sectional descriptive study was conducted in the Faculty of Medicine, University of Colombo in September 2013 with the participation of first and fourth year medical students and pre-intern medical graduates. Data was collected using a self administered, pre-tested questionnaire that collected data on socio-demographic details, intention to migrate and factors influencing a decision for or against migration.

**Results:**

There were 374 respondents, 162 from first year (females; 104, 64.2%), 159 from fourth year (females; 85, 53.5%) and 53 pre interns (females; 22, 41.5%). Of the entire sample, 89 (23.8%) had already decided to migrate while another 121 (32.3%) were not sure of their decision. The most cited reasons for migration were a perceived better quality of life, better earnings and more training opportunities in the host country. There were no socio-demographic characteristics that had a significant association with the intention to migrate, indicating that it is a highly individualized decision.

**Conclusions:**

The rate of intention to migrate in this sample is low when compared to international studies from Africa and South Asia, but is still significant. The core reasons which prompt doctors to migrate should be addressed by a multipronged approach to prevent brain drain.

## Background

Emigration of professionals, also known as ‘brain drain’, is a well recognized social phenomenon in Sri Lanka, particularly among health professionals. Up to 2009, 11% of board certified medical specialists have migrated from Sri Lanka before the completion of the period of bond (statutory service duration in lieu for the paid leave obtained for further studies) with Ministry of Health, Sri Lanka [1]. When nearly one in ten specialists leaves the country, a significant impact on the health care provision system is inevitable. This is confirmed by the ratio of serving doctors to available cadre positions. While the discrepancy is less in more affluent and cosmopolitan areas of the country such as the Western province where the capital Colombo is located, it is glaringly unsatisfactory in more remote Northern and Eastern provinces [2]. Doctors emigrate at various points post-qualification, however there have been no studies providing data on the migration of other non-specialist doctors out of Sri Lanka. Nonetheless, this unaccounted for “brain drain” also significantly affects the health care system.

It has been postulated that the most motivated and distinguished professionals among peer groups are the first to migrate. They are the individuals who are most likely to be in demand to assume positions in leadership and academia in clinical services, educational programs and research activities. Their migration is a loss to the country of their origin [3]. Sri Lanka is a middle income country, with just 4% of its GDP dedicated to health. Free education is provided to all, and the state invests considerable amounts of money and resources in training doctors; thus the economic impact resulting from migration of doctors is considerable.

In this context it is crucial for a developing country to develop strategies to minimize the emigration of health professionals. Though restrictions to leave the country and bonds between state and professionals can contribute to stem the exodus, the quality of service provided by them in a state of dissatisfaction cannot be assured of. Hence it is of prime importance to identify factors that make professionals wanting to migrate and rectify them so that root cause of the issue is eliminated.

The Faculty of Medicine of the University of Colombo is the oldest medical school in Sri Lanka, and produces approximately 200 medical graduates each year (approximately 25% of medical graduates from state universities in an academic year). This study was conducted to: a) identify the proportion of medical graduates and undergraduates from Faculty of Medicine, University of Colombo intending to migrate away from Sri Lanka and b) identify factors contributing for intention to stay or migrate.

## Methods

A cross sectional descriptive study was conducted in the Faculty of Medicine, University of Colombo in September 2013 with the participation of first and fourth year medical students and pre-intern medical graduates (medical graduates awaiting their internship after completing their undergraduate degree). All students/graduates in the eligible categories were invited to participate. First and fourth year medical students were selected to represent the extremes of medical students passage through the medical school. The pre-intern period is where the new graduates are first exposed to a real professional environment with opportunities to associate with seniors. These inputs may act as a determining factor in decision making for the future.

Data was collected using a self-administered questionnaire which was designed by the investigators following an extensive literature search. It was pre-tested on 10 students from a batch that was not a part of the study sample. The first section of the questionnaire was on socio-demographic factors, details of academic achievements and intention to migrate abroad to which they had to answer ‘yes’, ‘no’ and ‘not sure’. Based on the answer to the last question they were directed to answer only one of the next three sections. These sections were mainly on participants’ reasons to stay in the country or migrate and factors related to the decision. Each section had questions regarding prevailing factors supporting a decision to migrate, as well those opposing it. First and fourth year students were approached after a lecture session and invited to participate in the study. Those consenting were given the questionnaire. Pre-interns were sent the same questionnaire developed as a Google^®^ form sent to their e-mail address. They were requested to fill and submit the form if they consent to participate in the study.

Data were entered and analyzed using Statistical Package of Social Sciences (SPSS)^®^ version 19. Descriptive statistics were outlined with frequencies, proportions, percentages and summarized using mean with standard deviation. Significance of dichotomous variables were analyzed using chi-square test (and Fishers exact test where appropriate), and those of continuous variables with independent t-test. Ethical approval for the study was obtained from Ethics Review Committee, Faculty of Medicine, University of Colombo.

## Results

There were 374 respondents for the study, 162 first year students (Response rate; 84%, females; 104, 64.2%), 159 fourth year students (Response rate; 84.6%, females; 85, 53.5%) and 53 pre-interns (Response rate; 42% females; 22, 41.5%). The highest percentage of participants (37.7%) were from Colombo district where the faculty was located within the precincts of the capital city, Colombo. Out of all the participants 84.8% were Sinhalese and 11.5% were Tamils. Two participants each from first and fourth year batches had not responded to the question on intention to migrate. The basic demographic characteristics of the sample are summarized in Table 1 and Intention on migration of the participants is shown in the Table 2.

**Table 1:** **Characteristics of the sample (n = 374)**

Category	Number/mean ± SD	Percentage
**Gender**		
Male	163	43.6
Female	211	56.4
**Age (in years)**	23.35 ± 1.97 (range 20–28)	
**Year of study**		
First year	162	43.3
Fourth year	159	42.5
Pre-intern	53	14.2
**Ethnicity**		
Sinhala	317	84.8
Tamil	43	11.5
Muslim	12	3.2
Other	1	0.3
**District of residence**		
Within Colombo	141	37.7
Outside Colombo	233	62.3
**Family income (in rupees, 100 rupees approximates 1 USD)**		
<20,000	48	19.3
20,001 – 40,000	91	43.6
40,001 – 100,000	156	41.7
>100,000	55	14.7
Not answered	24	6.4
**Having own income**		
Yes	47	12.6
No	204	54.5
Not answered	123	32.8

**Table 2:** Expressed intention to migrate among the participants of the study*

Intention to migrate	First year (percentage)	Fourth year (percentage)	Pre-intern (percentage)	Total (percentage)
Yes	41 (11)	30 (8)	18 (4.8)	89 (23.8)
No	61 (16.4)	79 (21.2)	22 (6)	162 (43.6)
Not sure	59 (15.9)	49 (13.2)	13 (3.5)	121 (32.6)
Total (columns)	161 (43.3)	158 (42.4)	53 (14.3)	372

*All percentages are calculated using the grand total (n-372) as the denominator.

Table 3 shows several potential associations that may influence the intention to migrate. However, in our sample none of the variables (gender, ethnicity, family income, individual income, having relatives abroad, intention to do postgraduate studies) predicted the intention to migrate (p < 0.05).

**Table 3:** **Association between intention to migrate and potential deciding factors**

Variable	Intending to migrate	Not intending to migrate	Chi square value (p value–two tailed)
Gender			
Male	47	72	1.61 (0.24)
Female	42	90	
Ethnicity			
Sinhalese	75	131	5.26 (0.07)
Tamil	8	24	
Muslim	5	7	
Having relatives/close friends abroad			
Yes	43	72	0.35 (0.55)
No	46	90	
Year of study			
First year	41	61	5.59 (0.06)
Fourth year	30	79	
Pre-intern (graduate)	18	22	
Ever visited or stayed abroad			
Yes	21	53	2.30 (0.13)
No	68	109	
Intending to do postgraduate studies			
Yes	54	90	0.61 (0.43)
No/Not sure	35	72	
Having an individual income			
Yes	22	25	3.25 (0.07)
No	67	137	

### Participants with intention to migrate (n = 89)

The preferred countries for migration were Australia, the United Kingdom and the United States of America with 64 (71.9%), 60 (67.4%) and 47 (52.8%) participants selecting these countries as a potential country for migration respectively. However, of the entire sample, only one had sat for a selection exam to be eligible to practice in one of those countries (i.e., the Australian Medical Council Examination for foreign graduates). Thirty one (34.8%) intended to migrate after entering a postgraduate training programme in Sri Lanka while 22 (24.7%) and 19 (21.3%) intended to migrate soon after internship or after few years of local practice without postgraduate training. Six (6.7%) were planning to migrate even before internship which would effectively disqualify them from Sri Lanka Medical Council permanent registration. The majority (46–51.7%) expressed the wish stay abroad for less than 10 years while 22 (24.7%) wanted to settle abroad for good. Eighteen (20.2%) wanted to stay for more than 10 years but return to Sri Lanka.

Those intending to migrate were asked to name the reasons for wanting to do so. The three most common answers to these questions were: presumed better quality of life (68.97%); need for a higher income (48.28%); and availability of better medical services (29.89%). Participants also marked the positive effects of staying in Sri Lanka despite their wish to migrate. The three leading reasons in this regard as perceived by the participants were: opportunity to stay with the family (71.19%); opportunity to serve the country (64.41%); and being comfortable with the local setting (55.93%). Twenty five have noted obstacles to their intention to migrate, and the main obstacle mentioned was lack of finances (32%).

### Participants with no intention to migrate (n = 162)

In this group, 28 (17.3%) were initially intending to migrate and changed their intention later on. The reasons for them to stay in the country were: need to stay with family (86.75%); intention to serve country (69.54%); and being comfortable with the local setting (60.93%). The following were the positive effects of migration identified by the participants despite their intention to not to migrate: better income (76.76%); better quality of life (66.2%); and better postgraduate training abroad (30.28%).

### Participants who were not sure about the intention to migrate (n = 121)

A considerable proportion (i.e., 32.4%) of the sample was undecided on migration. The three topmost positive effects of migration as perceived by this group were: better quality of life (67.71%); better income (58.83%); and better political climate (27.08%). The three topmost reasons why they would consider staying in Sri Lanka were: need to stay with the family (85.59%); need to serve the country (72.07%); and being comfortable with the local setting (52.25%).

The factors favouring staying in home country and those favouring migration for each of the above three categories are listed in Table 4 in order of priority.

**Table 4:** **Topmost factors for and against migration in the sample**

Category	For migration (in order of priority)	Against migration (in order of priority)
Decided to migrate	Better quality of life	Family ties
	Better pay	Serve the country
	Better medical services	Familiarity with local setting
Decided not to migrate	Better pay	Family ties
	Better quality of life	Serve the country
	Better postgraduate training abroad	Familiarity with local setting
Not sure of decision to migrate	Better quality of life	Family ties
	Better pay	Serve the country
	Political stability and peace	Familiarity with local setting

## Discussion

This is the first study that has looked in to the aspirations of migration of medical graduates in Sri Lanka. It shows that a significant proportion of the sample (23.8%) intend to migrate while a larger percentage (32.4%) are still not sure of their ultimate decision. As mentioned previously, though data is not available for all locally trained doctors, 11% of board certified consultants have either not returned from their foreign training or have left the country after coming back [1]. Therefore the risk of brain drain is real and still has the potential to amplify in the coming years with larger numbers of locally trained graduates intending to leave the country.

However, it is notable that of the socio-demographic and situational factors that were analyzed, none were significantly associated with an intention to migrate (table 3). This means that the subgroup that is intending to migrate cannot be differentiated from others based on any predictive traits. The decision to migrate therefore may be a highly individualized decision fuelled by unique and heterogenous contributory factors. However, when queried about why they intended to migrate most seemed to cite a better quality of life and better earnings as the prime cause for emigration. Even those not intending to migrate immediately seem to think along those lines. In the days of global connectivity, the salaries and life styles of peers abroad is easily visible to undergraduates in Sri Lanka and communication with them is always easier than ever before. Therefore, people do have ways and means of appreciating the cost of living, salaries and savings in a foreign country much better than before. It is also noted that most undergraduates responded that one reason they would like to stay behind is to be with their family. However, again in view of ease of communication, this may be less of a limiting factor now, as effective means of communication over the internet can maintain close connectivity across borders.

Local data on the topic of migration of medical professionals is limited. A single qualitative study from Sri Lanka on migration of medical specialists has revealed better quality of life, having to work in rural parts of Sri Lanka if they remain in the country, future career development, and social security as topmost reasons to migrate [1]. This data is from only 5 emigrated specialists as the study had a poor respondent rate. Hence the generalisability of this data is questionable. Our study is unique as we look at factors that influence a decision to migrate before the act of actual migration.

It is also worthwhile to look at similar studies from other developing nations for purposes of comparison. Sub-Saharan Africa is one of the worst affected regions of healthcare worker migration [4]. This region shares 25% of global disease burden but has only 3% of world’s health care workers [5]. A study from Ethiopia looked at intention to migrate among medical students and reported that 53% of the participants intended to migrate [6]. Unlike in our study they found that male students and senior students had a significantly higher percentage of those willing to migrate. This difference could be due to different socio-economic backgrounds seen in the two countries. Both countries were tormented by civil unrest but Sri Lanka has emerged from a civil war to a peaceful country since 2009. The country’s gross national product is on the rise and it is attracting more foreign investments with improved standards of living. Therefore many professionals who were intending to leave may have better reasons to stay at home now. A review on sub-Saharan Africa’s health care worker migration problem found push factors (factors favouring emigration) such as low public sector salaries, demoralizing work conditions and political instability to be the major driving forces for the loss of professionals [5].

In South Asia, A study from Pakistan [3] has recognized poor salary structure and poor quality of training as the two commonest reasons that final year medical students were considering as reasons for emigration. It was also noted that in two universities included in the study, over 65% and 95% of students were intending to migrate. Poor work environment was also a major contributory factor for emigration. Another Pakistani study has found lucrative salary abroad, quality of training, job satisfaction and better way of life as common reasons identified by medical students who intend to migrate [7]. They also noted terrorism and violence against doctors as other contributing factors. Similar reasons have been cited in other studies conducted in developing nations [6, 8–10]. The plight of developing nations in keeping their trained professionals is best demonstrated by a study in Nepal where 723 graduates from its first medical school, Institute of Medicine, Nepal, was traced for their working station at least 6 years after graduation. The cohort of doctors traced included graduates of 22 classes from 1983–2004. Of the total number (723), 710 doctors were traced and a staggering 36% of them (256 doctors) were working overseas on a permanent basis [11]. The largest proportion of them (188, 26.5%) were in the United States.

A study from South Pacific islands of Tonga, Fiji and Samoa (n=251) showed that both income and non-income factors were important in the decision to migrate among doctors and nurses. Those choosing to migrate were more likely to have relatives living in the recipient country, and being exposed to superior working conditions in that country before deciding to migrate [12, 13].

A review on the topic found that two countries; India and Indonesia (notably two very populous countries as well), to be the most affected by emigration of medical professionals in Asia [14]. In addition to describing the commonly observed reasons for emigration, the study also points out that the exodus of medical professionals leaves a void in the donor country that is unequally distributed in different geographical regions with the more poor and remote areas of the country being more neglected with respect to medical needs. The same fact is true in Sri Lanka as demonstrated in the cadre vacancies for medical specialists and non-specialist medical officers in districts far away from the capital, Colombo. In some countries, it may also affect the balance of private and public health care systems with those staying behind preferring one of these systems to render their services leaving the other under-staffed [15].

Migration of medical doctors is not a phenomenon observed in low and middle income countries only [16, 17]. Even in countries like South Africa where quality of medical training and general income for professionals is better than in low income countries, migration of doctors is still a problem. In a study of migrant doctors from South Africa who were permanently employed in other countries, 75% of them encouraged young doctors to migrate citing reasons such as better pay, better training in the recipient country and disadvantaged social circumstances such as HIV/AIDS epidemic and high crime rate in donor country [16]. However, the sample size was small in this study (n=29) to draw generalized conclusions about the overall migrant population of doctors from South Africa. In the European union, after its expansion to include new member states from Eastern Europe, migration of doctors from East to West was observed though not in large numbers as initially expected. Even within the EU, the income disparity was quite large with a general practitioner in France earning approximately ten times more than one in Romania in 2009 [18]. Similarly, in the South East Asian region, Singapore and Malaysia have become the largest importers of professionals while Philippines and Indonesia are the biggest exporters [15]. Overall, on a global scale, United States, United Kingdom, Australia and Canada are the most attractive options for migrating doctors as shown by the statistics of cross border migration of professionals [19].

Many international studies from African and South Asian developing countries record a higher percentage of an intention to migrate than in our study [16, 20, 21]. This may partly be due to the fact that Sri Lanka, unlike some of its trouble-ridden neighbors, has emerged free of domestic unrest and is looking towards political stability and economic growth in years to come. It is also possible that in some of the previously quoted studies, the intention to migrate is overinflated as the surveyors have not offered a “not sure” option to students when asking about their intention to stay or migrate. Another reason why the rates are low may be due to the fact that Sri Lanka has well established postgraduate training programmes that are internationally accredited. Nevertheless it must be appreciated that a significant percentage of locally trained graduates still intend to leave the country for a perceived better quality of life, better income and training opportunities. As pointed out previously, keeping them in the country by means of the legal restrictions is not a useful solution as it will lead to discontent with circumstances of service. The disparity between earnings in Sri Lanka and developed countries is huge; for example, a newly qualified specialist employed in the state sector will earn around USD 12000 per annum. An equivalent specialist in the UK will receive a base salary of USD 120 000, i.e., ten times what they would earn in Sri Lanka. However, most Sri Lankan specialists engage in part time private medical practice, which augments their income considerably, albeit at great cost to personal time and leisure. The long term solution would be to standardize the medical service with regard to pay, training opportunities and workload to match that of developing countries to which local graduates yearn to emigrate [22]. A systematic review on the topic has also suggested motivation building endevours such as financial rewards, career development opportunities, continuous professional education, better hospital infrastructure/resources and improvements in overall hospital management as ways to stall the migration of professionals [23]. It is a time consuming long process but would be beneficial to the country on the long run as training of medical undergraduates and postgraduates is currently entirely sponsored by the state, and each person leaving is an enormous loss of an investment to the system.

There were several limitations in this study. One was the poor response rate from pre-interns. They were located in different parts of the country and attempts to contact them through a Google^®^ form did not give a satisfactory response rate. This study was performed in a selected medical faulty of the country which has six other medical faculties. The wishes and plans of our sample could significantly differ from the others, particularly because the selected faculty is located in the capital city while the others are in different parts of the country. To make meaningful interpretation on this data it is important to have statistics on migration of Sri Lankan medical professionals accurately which is currently not available to the best of our knowledge. It is recommended that further studies of qualitative nature are carried out among medical undergraduates, postgraduates and practicing medical officers to gain a deeper understanding of the mechanics and aspirations of migration.

## Conclusion

This cross-sectional survey from the largest medical faculty of Sri Lanka showed that out of a sample of 374 first year, fourth year students and newly passed out graduates, 23.8% have already decided to migrate while another 32.3% were unsure as to whether they would choose to migrate or not. The most cited reasons for migration were a perceived better quality of life, better earnings and more training opportunities in the host country. The most commonly cited reason against migration was the loss of family bonds. There were no socio-demographic characteristics that had a significant association with an intention to migrate in this sample indicating that it is a highly personal and an individualized decision without any group characteristics. The rates of intention to migrate are low when compared to international studies from Africa and South Asia but nevertheless significant. Observing the trends of migration, maintaining accurate databases of migrating medical professionals and having a multi sectoral policy plan to uplift the quality of life of medical professionals is a need of the hour to curb the “brain drain”.

## Abbreviations

USD: United States Dollar.
